# STAKEHOLDER ENGAGEMENT FOR THE DESIGN OF GENERATIVE AI TOOLS: INCLUSIVE DESIGN APPROACHES

**DOI:** 10.1093/geroni/igae098.1918

**Published:** 2024-12-31

**Authors:** George Demiris

**Affiliations:** University of Pennsylvania, Philadelphia, Pennsylvania, United States

## Abstract

We present an approach to active and ongoing stakeholder engagement throughout the design lifecycle of generative AI applications. We highlight case studies from the Penn Artificial Intelligence and Technology Collaboratory for Healthy Aging (PennnAITech) of active involvement of older adults, family members, clinicians and social services coordinators as stakeholders in the early phases of design and prototype testing and demonstrate how this participatory approach can address not only usability challenges, but maximize the accessibility and relevance of Generative AI for older adult users. One example includes an NSF funded study using Large Language Model (LLM) tools to address social isolation and loneliness for community dwelling older adults living alone. In this project, older adults were invited to serve as co-designers of the LLM blueprint, its functionalities and purposes. Discussions around transparency and disclosure informed the implementation approach. The second example explores the use of Generative AI for a fall prevention program that captures fall risk scores using depth sensing. This project targets community dwelling older adults with mild cognitive impairment who live in low resource settings. The use of Generative AI is meant to make the fall risk information more accessible and actionable for older adults and their families. Co-design sessions provided system specifications for a chatbot that serves as a fall risk coach. As Generative AI applications continue to expand, assuring that we center older adults’ voices as part of an inclusive design effort will become critical for these systems to be accessible and relevant for their end users.

